# Cross‐Sectional Analysis of Metabolic Tumor Burden Detected by F‐18 FDG PET/CT and Circulating Tumor DNA in Advanced Breast Cancer

**DOI:** 10.1002/cam4.71049

**Published:** 2025-08-18

**Authors:** Recep Halit Tokac, Ekin Cemre Bayram Tokac, Ulkem Yararbas, Ayca Aykut, Asude Durmaz, Haluk Akin, Aziz Murat Argon

**Affiliations:** ^1^ Department of Nuclear Medicine Ege University Faculty of Medicine Izmir Turkey; ^2^ Department of Medical Genetics Ege University Faculty of Medicine Izmir Turkey

**Keywords:** breast cancer, metabolic tumor burden, total alteration number in ctDNA, variant allele frequency (VAF)

## Abstract

**Background:**

This study investigated the relationship between circulating tumor DNA (ctDNA) parameters and metabolic parameters from FDG PET/CT in advanced breast cancer (ABC) patients.

**Methods:**

In this retrospective single‐center study, 47 ABC patients who underwent both liquid biopsy and FDG PET/CT were analyzed.

**Results:**

Results showed that 27 patients (57.4%) had detectable ctDNA. Patients with detectable ctDNA demonstrated significantly higher whole‐body metabolic tumor volume (WB‐MTV) (*p* = 0.002) and whole‐body total lesion glycolysis (WB‐TLG) (*p* = 0.006) compared to those without ctDNA, while no significant difference was found in SUVmax or SUVmean. A moderate correlation was observed between variant allele frequency (VAF) values and metabolic parameters: maximum VAF correlated with SUVmax, WB‐MTV, and WB‐TLG (*r* = 0.407, *p* = 0.005; *r* = 0.457, *p* = 0.001; *r* = 0.415, *p* = 0.004, respectively). Mean VAF correlated with SUVmax, WB‐MTV, and WB‐TLG (*r* = 0.406, *p* = 0.005; *r* = 0.446, *p* = 0.002; *r* = 0.404, *p* = 0.005, respectively). The total VAF correlated with SUVmax, WB‐MTV, and WB‐TLG (*r* = 0.394, *p* = 0.006; *r* = 0.465, *p* = 0.001; *r* = 0.430, *p* = 0.003, respectively). When excluding patients without detectable ctDNA, the correlation between VAF values and WB‐MTV, WB‐TLG disappeared, while the correlation with SUVmax persisted. Total alteration number in ctDNA showed a moderate correlation with WB‐MTV and WB‐TLG (*r* = 0.563, *p* < 0.001; *r* = 0.459, *p* = 0.001, respectively). Correlation with WB‐MTV remained significant when excluding patients without detectable ctDNA (*r* = 0.500, *p* = 0.008).

**Conclusions:**

These findings suggest that metabolic tumor burden correlates with ctDNA detection and characteristics, potentially offering complementary information for disease monitoring, treatment selection, and response assessment in ABC. The combined use of these parameters may improve prognostic evaluation and guide personalized treatment strategies.

**Abbreviations**
ABC: advanced‐stage breast cancer
cfDNA: cell‐free DNA
CNVs: copy number variations
CT: computed tomography
CTC: circulating tumor cell
ctDNA: cell‐tumor DNA
EVs: extracellular vesicles
FDG: [^18^F]Fluorodeoxyglucose
HR: hormone receptor
MIP: maximum intensity projection
MRI: magnetic resonance imaging
PET: positron emission tomography
SNVs: single‐nucleotide variations
SUVmax: maximum standardized uptake value
SUVmean: mean standardized uptake value
TN: triple negative
VAF: variant allele frequency
VUS: variants of uncertain significance
WB‐MTV: whole‐body metabolic tumor volume
WB‐TLG: whole‐body total lesion glycolysis

## 1 Introduction

According to a global cancer epidemiology study conducted in 2022, breast cancer in women is the most frequently diagnosed cancer worldwide and ranks as the fourth leading cause of cancer‐related mortality among all cancers [1]. The survival rate in breast cancer varies depending on the stage. While the five‐year survival rate for localized breast cancer without nodal metastasis is 99%, this rate drops to 27% in the presence of distant metastasis [2].

There is a clear need for increased knowledge of new prognostic and predictive biomarkers in breast cancer. This information is critically important to assist clinicians in diagnosis, risk classification, identification of disease subtypes, prediction of treatment response, and monitoring to facilitate the management of both primary and metastatic breast cancer in a personalized manner [3]. In recent years, molecular profiling has been guiding clinicians in these areas [4]. The inclusion of additional tumor characteristics based on imaging and molecular parameters can provide more precise information on prognosis in cancers such as breast cancer, which could lead to improved treatment options and appropriate follow‐up plans [5].

Liquid biopsy, one of the molecular profiling methods, has become a prominent topic in recent years due to its advantages, such as ease of obtaining material, providing comprehensive information in metastatic disease, real‐time data provision, and prognostic assessment [6]. Circulating tumor DNA (ctDNA) obtained from liquid biopsy in advanced‐stage breast cancer (ABC) can overcome the costs, limitations, and difficulties in the identification, extraction, and characterization of circulating tumor cells (CTCs) and extracellular vesicles (EVs). Recently, numerous technologies and commercial assays have been rapidly developed to detect ctDNA, and studies investigating the potential role of this biomarker have been published [7, 8]. Specific mutations suitable for molecular targeting can be identified with ctDNA, and parameters that enable prognosis determination have been recognized. One such parameter is variant allele frequency (VAF) [9]. VAF represents the proportion of the total number of alterations in ctDNA at a specific locus in the genome compared to the same locus in normal tissue. A high VAF value has been observed to be associated with poor survival in various cancers [10]. Additionally, a high total number of alterations in ctDNA indicates increased tumor heterogeneity, which has been shown to correlate with poor prognosis [11].

The routine imaging method used in the diagnosis, staging, and follow‐up of breast cancer is PET/CT scanning with 18F‐FDG, a radiolabeled glucose analog that accumulates in tissues with high glucose metabolism [12]. In routine practice, the uptake of FDG in a tissue is evaluated using SUVmax. Additionally, other quantitative parameters, such as SUVmean and whole‐body metabolic tumor volume (WB‐MTV), and whole‐body total lesion glycolysis (WB‐TLG), which are formed by the product of these two quantitative parameters, have been shown in studies to be potential prognostic markers for survival in breast cancer [13, 14, 15] Volumetric parameters like TLG and MTV more accurately represent tumor burden because they consider the metabolic activity of the disease, rather than just the tumor burden assessed through CT or MRI scans. In the literature, studies have demonstrated the relationship between the presence of detectable ctDNA and its parameters that reflect the ratio of ctDNA to normal cell loci, with metabolic parameters detected by FDG PET/CT in cancers such as lung, head and neck cancers, and lymphoma [16, 17, 18, 19].

In this study, we aim to retrospectively examine the relationship between ctDNA presence and its parameters (maximum VAF, mean VAF, total VAF, total number of alterations in ctDNA) determined by liquid biopsy, and metabolic parameters (SUVmax, SUVmean, WB‐MTV, WB‐TLG) obtained from whole‐body F‐18 FDG PET/CT scans in ABC cancer patients. This study aims to contribute to the literature by improving prognosis determination and the use of testing tools in ABC patients.

## 2 Material and Methods

### 2.1 Patient Selection and Study Design

This study was conducted as a single‐center retrospective study at Ege University Faculty of Medicine between September 2021 and December 2023. Patients included in the study were those previously diagnosed with breast cancer and who, after receiving necessary treatments, experienced disease progression during follow‐up and were evaluated at Ege University for treatment modification in ABC. The inclusion criteria for the study are as follows: (1) Patients who had undergone liquid biopsy testing and FDG PET/CT scanning within a short time frame (0–60 days), (2) No radiotherapy and/or chemotherapy administered between the liquid biopsy and FDG PET/CT scans, (3) Presence of measurable hypermetabolic lesions in the FDG PET/CT scan, (4) Complete patient data is available in the archival records. The exclusion criteria are as follows: (1) A time gap of more than 60 days between the liquid biopsy and FDG PET/CT scans, (2) Radiotherapy and/or chemotherapy administered between the liquid biopsy and FDG PET/CT scans, (3) Presence of a second primary malignancy, (4) Absence of measurable hypermetabolic lesions in the FDG PET/CT scan, (5) Incomplete archival data. Patients whose data were to be used for this research were included in the study in accordance with the principles of the Declaration of Helsinki, after obtaining approval from the Ethics Committee for Medical Research at Ege University (Approval No: 24‐7T/34). Written consent was obtained for the use of the data of the included patients. The data of patients who did not provide consent were excluded from the study.

### 2.2 Liquid Biopsy Collection and Cell‐Free DNA Extraction

**FIGURE 1:**
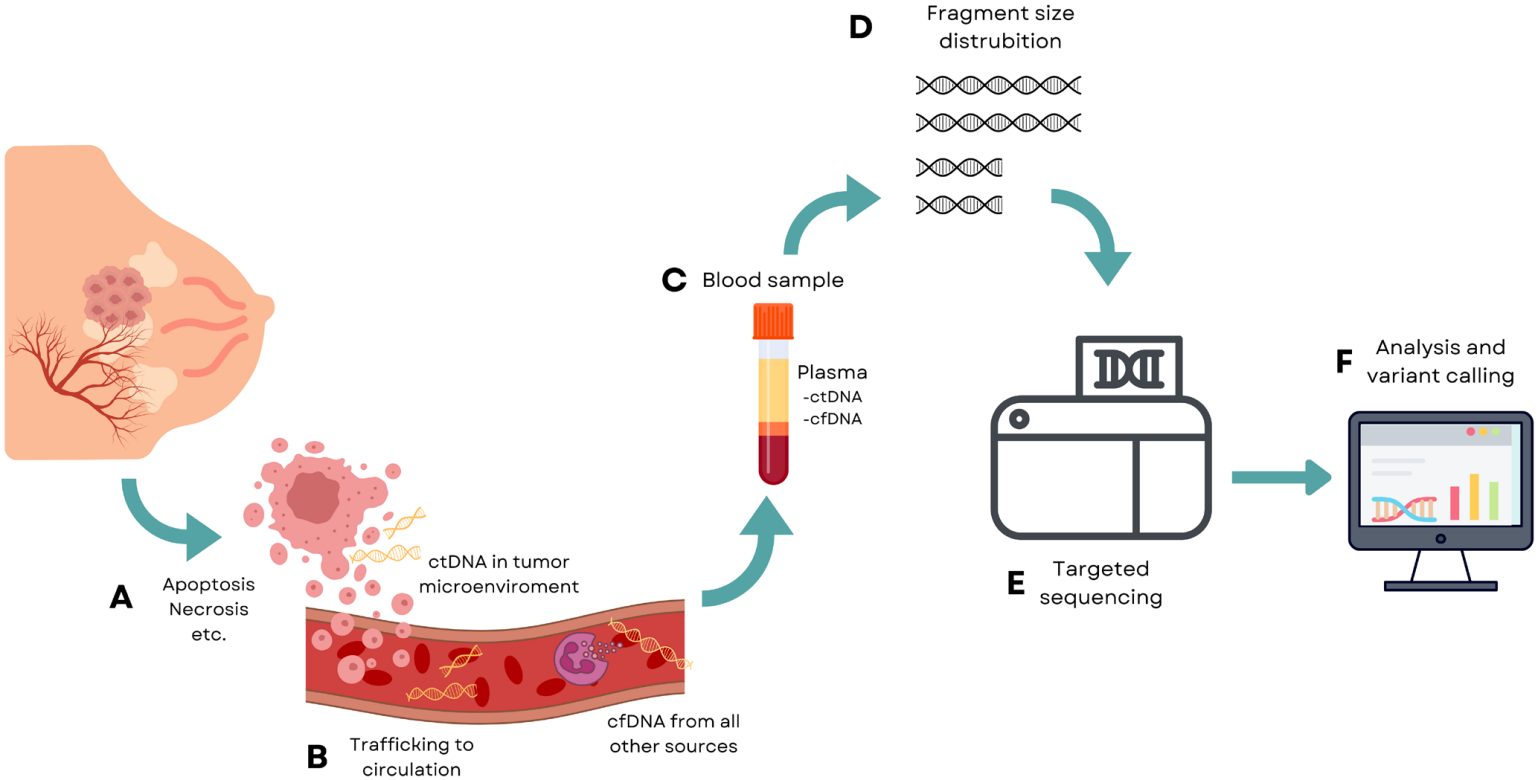
Tumor cells release genetic material into the bloodstream through various cellular processes, including cell death and active shedding (A). These genetic fragments detach from the primary tumor and enter the circulatory system (B). Whole blood samples are centrifuged to separate plasma, which is enriched in circulating tumor DNA (ctDNA) and cell‐free DNA (cfDNA), unlike the buffy coat, which contains predominantly circulating hematologic cells (C). cfDNA and ctDNA are then isolated from the processed plasma samples, and fragment size distribution is analyzed to prepare for sequencing of ctDNA (D). A 77‐gene panel was utilized for sequencing, as detailed in Table 1 (E). Variant calling and analysis were performed using AVENIO Oncology Analysis Software (Version 2.0.0) and NAVIFY Mutation Profiler (Version 2.1.0) (F).

Blood samples were collected in Roche Cell‐Free DNA (cfDNA) Collection tubes and processed within 2 h of collection. For plasma isolation, whole blood was centrifuged at 500 *g* for 10 min within 2 h of collection and visually inspected for hemolysis, with severely hemolyzed samples excluded to prevent leukocyte DNA contamination. Processed plasma was stored at −80°C. Prior to DNA extraction, plasma samples were thawed and centrifuged at 1800× *g* for 5 min to remove cellular debris. Four milliliters of the clarified plasma were transferred to a clean tube. Cell‐free DNA was extracted using the AVENIO cfDNA Isolation Kit (Roche Diagnostics) following the manufacturer's protocol, with final elution in 65 μL of elution buffer (Figure 1).

### 2.3 Checking the Quality of Plasma‐Extracted cfDNA

Plasma‐extracted cfDNA was quantified using fluorometry with a Qubit dsDNA HS Assay Kit (Thermo Fisher Scientific). Sample quality and DNA integrity were assessed via capillary electrophoresis using the Agilent High Sensitivity DNA Kit on a 2100 Bioanalyzer. The ratio of short to long DNA fragments was calculated to evaluate potential genomic DNA contamination. All cfDNA samples met the quality criteria to be included. They display a dominant peak at 160–180 bp and minor peaks around 320–480 bp. The absence of large fragments (> 1000 bp) confirmed minimal genomic DNA contamination, ensuring suitability for downstream applications such as sequencing. Total isolated DNA mass was calculated using the formula:

$$\text{Isolated}\;\mathrm{DNA}\;\text{Mass}\;\left(\mathrm{ng}\right)=\text{cfDNA Concentration}\;\left(\mathrm{ng}/\mathrm{\mu L}\right)\times 65\;\mathrm{\mu L}$$

### 2.4 Preparing Sequencing Libraries and Target Enrichment

Library preparation was performed according to the manufacturer's protocol using the AVENIO ctDNA Library Prep Kit V2 (Roche Diagnostics). Each sample was prepared with 50 ng of cfDNA as input material, though in cases of limited availability, a minimum of 10 ng was used. Adapter ligation was carried out with the addition of 10 μL of unique sample adapters. Target enrichment was performed via overnight hybridization (16–18 h) using the AVENIO ctDNA Enrichment Kit V2. Following hybridization cleanup, PCR amplification of the enriched ctDNA, and a final cleanup of the post‐capture PCR product were steps performed according to the producer's manual. This workflow enabled detection of single‐nucleotide variations (SNVs), gene fusions, and copy number variations (CNVs) in targeted regions (Table 1).

**TABLE 1:** The panel targeted the following genomic regions.

Category	Gene	Remarks
Single nucleotide variants (SNVs)	ABL1, AKT1, AKT2, ALK, APC, AR, ARAF, BRAF, BRCA1, BRCA2, CCND1, CCND2, CCND3, CD274, CDK4, CDK6, CDKN2A, CSF1R, CTNNB1, DDR2, DPYD, EGFR, ERBB2, ESR1, EZH2, FBXW7, FGFR1, FGFR2, FGFR3, FLT1, FLT3, FLT4, GATA3, GNA11, GNAQ, GNAS, IDH1, IDH2, JAK2, JAK3, KDR, KEAP1, KIT, KRAS, MAP2K1, MAP2K2, MET, MLH1, MSH2, MSH6, MTOR, NF2, NFE2L2, NRAS, NTRK1, PDCD1LG2, PDGFRA, PDGFRB, PIK3CA, PIK3R1, PMS2, PTCH1, PTEN, RAF1, RB1, RET, RNF43, ROS1, SMAD4, SMO, STK11, TP53, TERT promoter, TSC1, TSC2, UGT1A1, VHL	Coding regions of genes marked with an asterisk (*) were fully analyzed. For other genes, hotspot‐targeted sequencing was performed
Fusions	ALK, RET, ROS1, NTRK1, FGFR2, FGFR3	
Copy number variations (CNVs)	EGFR, ERBB2 (HER‐2), MET	

### 2.5 Sequencing and Data Analysis

**FIGURE 2:**
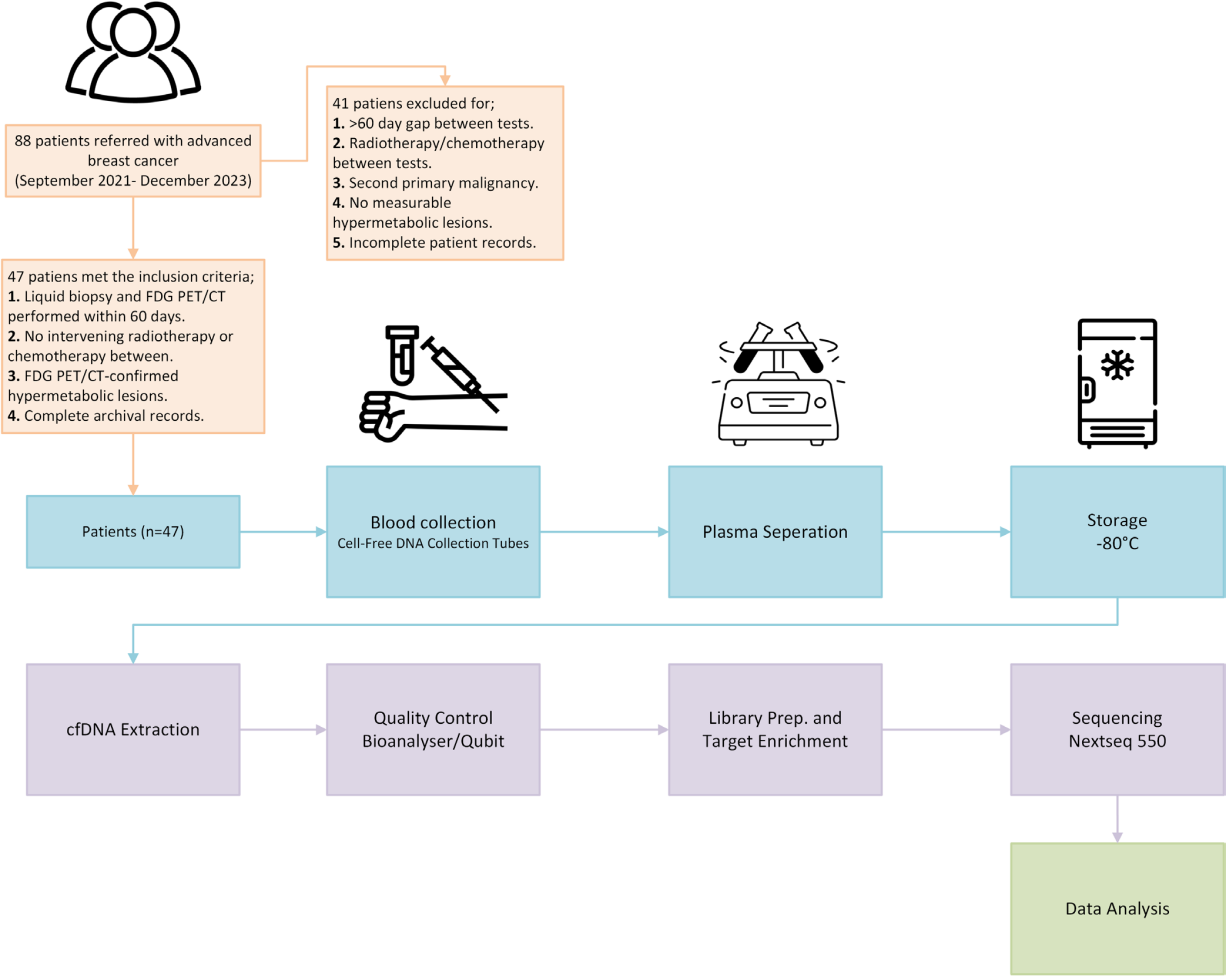
Liquid Biopsy Workflow Chart; Illustrates the key steps in the liquid biopsy process, including patient blood collection, sample storage, ctDNA extraction, and subsequent analysis.

Sequencing was performed on an Illumina NextSeq 550 platform (Illumina, San Diego, CA, USA) using the 300‐cycle NextSeq 500/550 Mid Output v2 kit in paired‐end mode (2 × 151 cycles). Sequences were aligned to the hg38 reference genome. Data analysis followed a multi‐step process: Initial data processing was performed using AVENIO Oncology Analysis Software (v2.0.0, Roche Diagnostics) with default parameter settings for the expanded panel. This included quality control assessment, variant calling based on a predefined “loci of interest” list, and initial variant filtering using the COSMIC, TCGA, ExAC, dbSNP, and 1000 Genomes databases (Figure 2). This pipeline enables the detection of low‐frequency alleles, down to 0.1%. The software generates three default reports for each sample in PDF format. Which included: a sample metrics report, a comprehensive variant report listing all unfiltered variants, and a refined variant report, where Roche's default criteria were applied for filtering. To prioritize actionable findings, unfiltered variants were cross‐referenced with disease‐specific databases (COSMIC, VARSOME, OncoKB, etc.) and classified according to AMP/ASCO/CAP guidelines. Germline variants were excluded and variants classified as pathogenic or likely pathogenic were prioritized for further investigation. Variants of uncertain significance (VUS) were annotated but deprioritized in downstream analyses. Clinical interpretation of variants is performed routinely using the CE‐IVD‐certified NAVIFY Mutation Profiler, with versions ranging from 2.1.0 to 2.3.2.c090e09, depending on the time of analysis. This software evaluated clinical relevance using multiple databases (COSMIC, TCGA, ClinVar, dbNSFP, CIViC, gnomAD, Mitelman, dbVar) and classified variants according to ASCO, AMP, and AACR guidelines into ESCAT tiers (ESMO Scale for Clinical Actionability of molecular Targets).

Tier 1‐A: Variants with approved therapies in professional guidelines.

Tier I‐B: Well‐powered studies with consensus from experts in the field.

Tier II‐C: Variants with approved therapies in other tumor types.

Tier II‐D: Variants supported by limited clinical/preclinical studies.

Tier III: Variants of uncertain significance (VUS) (not included).

Tier IV: Benign/likely benign variants (not included).

### 2.6 FDG PET/CT Imaging and Image Analysis

FDG PET/CT scans were performed after patients fasted for at least 6 h, with blood glucose levels below 200 mg/dL. Intravenous FDG was administered at approximately 3.7 MBq/kg, in accordance with international guidelines. After the IV injection, patients rested for 60 min without any physical activity. During this process, patients were encouraged to drink at least 1 L of water. Images were acquired in the supine position, covering the area from the vertex to the mid‐thigh, using a Siemens Biograph Truepoint 16 (Siemens Medical, Erlangen, Germany) device. For attenuation correction and anatomical correlation, low‐dose CT imaging was performed without the use of oral or IV contrast, with parameters set at 120 mA, 130 kV, a slice thickness of 5 mm, a rotation time of 0.6 s, a table speed of 1 mm/s, and a matrix size of 512 × 512. PET imaging was performed with an acquisition time of 2 min per bed position. The images obtained were evaluated on workstations using coronal, sagittal, and axial projections in PET, CT, and fusion PET/CT, along with maximum intensity projection (MIP) of the whole body. All measurable tumor lesions were selected for analysis. For each tumor lesion, the SUVmax, SUVmean, WB‐MTV, and WB‐TLG values were measured using the Siemens Syngo.via software. The SUVmax of a patient was defined as the highest SUVmax recorded among all detected lesions in the patient. MTV was calculated based on a 41% SUVmax threshold, and TLG was measured semi‐automatically for each lesion using the formula ‘SUVmean X MTV,’ with manual adjustments when necessary. The MTV and TLG values of all lesions were summed to obtain WB‐MTV and WB‐TLG. The whole‐body SUVmean was indirectly obtained using the formula WB‐TLG/WB‐MTV.

### 2.7 Statistical Analysis

Data analysis was performed using IBM SPSS Statistics for Windows, version 25. Descriptive statistics, including means and standard deviations, were calculated for categorical and numerical variables. The Kolmogorov–Smirnov and Shapiro–Wilk tests were used to assess the normality of numerical variables. Patients with at least one detected SNV in the liquid biopsy test were included in the ctDNA‐detected group. The relationship between WB‐MTV, WB‐TLG, SUVmax, SUVmean, and the presence or absence of ctDNA was evaluated using the Mann–Whitney *U* test due to the nonparametric nature of the data. In patients with multiple detected SNVs, the SNV with the highest VAF value was defined as the maximum VAF, the sum of the VAF values was defined as the total VAF, and the mean of all VAF values was defined as the mean VAF. The total alteration number in ctDNA was defined as the sum of all SNV and CNV numbers present in a case. The statistical relationship between maximum VAF, mean VAF, total VAF, and total alteration number in ctDNA with WB‐MTV, WB‐TLG, SUVmax, and SUVmean was evaluated using Spearman's Rho correlation, as these values did not follow a normal distribution. Additionally, descriptive statistics were calculated for the total alteration number in ctDNA within the ctDNA‐detected group. Patients without detected ctDNA were excluded, and the correlation between maximum VAF, mean VAF, total VAF, and the total change in ctDNA within the ctDNA‐positive group and metabolic parameters was further analyzed using Spearman's Rho correlation due to the non‐normal distribution of these variables. A confidence interval of 95% and a statistical significance level of *p* < 0.05 were considered for all tests.

## 3 Results

### 3.1 Patient Characteristics

**FIGURE 3:**
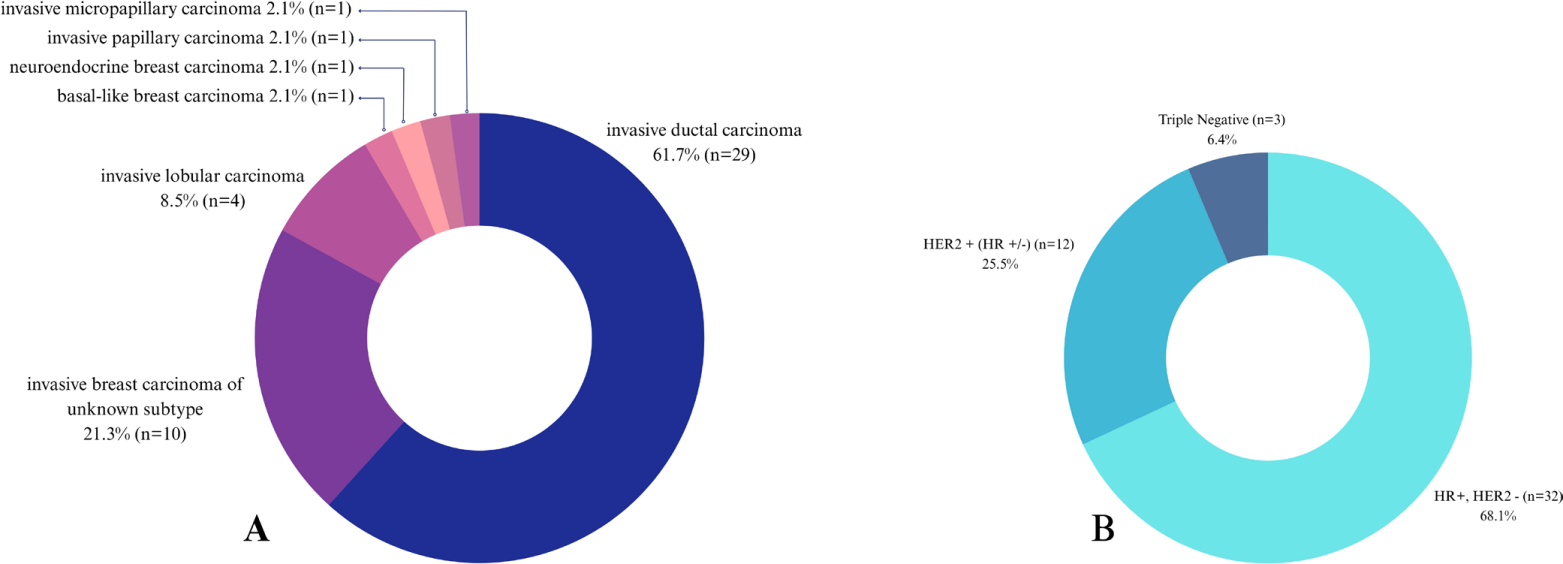
The pathology (A) and immunohistochemical analysis (B) results obtained after surgical treatment and true‐cut biopsy are presented as a pie chart.

Between September 2021 and December 2023, liquid biopsy tests were performed on 88 patients diagnosed with ABC at Ege University Faculty of Medicine. Of these, 47 patients who had recent FDG PET/CT scans and met the inclusion criteria were included in the study. All of the included patients were female, with an average age at diagnosis of 44.5 ± 10.4 years (range: 22–64). The average time between FDG PET/CT and liquid biopsy was 16.3 ± 14.5 days (range: 0–56). The metabolic parameters of the patients were evaluated, and the mean SUVmax was 13.64 ± 5.91 (range: 4.32–29.55), the mean SUVmean was 5.41 ± 2.21 (range: 2.04–10.48), the mean WB‐MTV was 150.28 ± 258.34 (range: 0.95–1444.39), and the mean WB‐TLG was 748.85 ± 1339.05 (range: 6.78–6589.57). The VAFmax of the patients was 9.14% ± 18.78% (range: 0%–83%), the mean VAF was 8.31% ± 17.19% (range: 0%–72%), and the total VAF was 12.31% ± 26.78% (range: 0%–144%). When patients without ctDNA were excluded, the average VAFmax was 15.91% ± 22.63% (range: 0.09%–83%), the mean VAF was 14.47% ± 20.75% (range: 0.09%–72%), and the total VAF was 21.44% ± 32.65% (range: 0.09%–144%). Pathological analysis following either true‐cut biopsy or surgery revealed invasive ductal carcinoma (IDC) in 29 patients (61.7%), invasive breast carcinoma of unknown subtype in 10 patients (21.3%), invasive lobular carcinoma (ILC) in 4 patients (8.5%), invasive micropapillary carcinoma in 1 patient (2.1%), invasive papillary carcinoma in 1 patient (2.1%), neuroendocrine breast carcinoma in 1 patient (2.1%), and basal‐like breast carcinoma in 1 patient (2.1%). Immunohistochemical evaluation revealed that 32 patients (68.1%) were hormone receptor‐positive/HER2‐negative (HR+/HER2‐), 12 patients (25.5%) were HER2‐positive (HER2+), and 3 patients (6.4%) were triple negative (TN) (Figure 3). When evaluating hypermetabolic metastatic sites, 37 patients (78.7%) had bone metastases, 23 patients (48.9%) had lymph node metastases, and 23 patients (48.9%) had visceral metastases (Table 2).

**TABLE 2:** Patient characteristics.

Characteristic	Value
Study period	September 2021–December 2023
Total patients tested	88
Patients meeting inclusion criteria	47
Average age (years)	44.5 ± 10.4 (Range: 22–64)
Average time between biopsy and PET/CT (days)	16.3 ± 14.5 (Range: 0–56)
Mean SUVmax	13.64 ± 5.91 (Range: 4.32–29.55)
Mean SUVmean	5.41 ± 2.21 (Range: 2.04–10.48)
Mean whole‐body MTV	150.28 ± 258.34 (Range: 0.95–1444.39)
Mean whole‐body TLG	748.85 ± 1339.05 (Range: 6.78–6589.57)
VAFmax	9.14% ± 18.78% (Range: 0%–83%)
Mean VAF	8.31% ± 17.19% (Range: 0%–72%)
Total VAF	12.31% ± 26.78% (Range: 0%–144%)
Hypermetabolic metastatic sites	Bone metastases: 37 patients (78.7%)
	Lymph node metastases: 23 patients (48.9%)
	Visceral metastases: 23 patients (48.9%)

### 3.2 ctDNA Alterations Detected by Liquid Biopsy

Of the 47 patients included in the study, 27 (57.4%) had detectable ctDNA, while 20 patients (42.6%) did not. Cases in which at least one SNV was detected in the liquid biopsy test and those SNVs were classified outside of Tier III (VUS) and Tier IV (considered benign or likely benign) were considered ctDNA‐positive. Additionally, 8 patients (17%) had at least one CNV, and these patients also had at least one SNV. None of the patients included in the study were found to have fusion gene alterations. Analysis of the alleles detected in the ctDNA revealed PIK3CA mutations in 11 patients (23.4%), TP53 mutations in 10 patients (21.3%), ESR1 mutations in 7 patients (14.9%), EGFR amplification in 6 patients (12.8%), MET amplification in 5 patients (10.6%), RB1 mutations in 3 patients (6.4%), ERBB2 amplification in 2 patients (4.2%), and one patient each (2.1%) with mutations in EGFR, JAK2, CCND3, AKT1, CDKN2A, KRAS, PTEN, PMS2, BRAF, and VHL. The subgroup analysis between metabolic tumor burden and immunohistochemical markers with SNV and CNV could not be performed due to the low number of patients in each group. However, the changes in immunohistochemical markers, VAFmax, mean VAF, and total VAF values in ctDNA, as well as metabolic tumor burden, are presented in Table S2.

### 3.3 Relationship Between ctDNA Presence and Metabolic Tumor Burden

Patients with detectable ctDNA had significantly higher WB‐MTV (*p* = 0.002) and WB‐TLG (*p* = 0.006) values compared to those without ctDNA. Additionally, no statistically significant relationship was found between the SUVmax (p: 0.061) and SUVmean (p: 0.651) values and the groups with and without detectable ctDNA (Table 3). Due to the detection of high VAF values in 5 patients, a hereditary cancer panel was evaluated. No germline mutations were found in 4 patients that could explain the elevated VAF values, and the hereditary cancer panel was not performed in 1 patient.

**TABLE 3:** Comparison between patients with detectable ctDNA and patients without detectable ctDNA.

	Patients with detectable ctDNA	Patients without detectable ctDNA	*p*
Number of patients	27	20	—
WB‐MTV*	224.06 ± 318.75 (12.63–1444.39)	50.68 ± 66.04 (0.95–271.66)	0.002*
WB‐TLG*	1131.83 ± 1661.06 (32.35–6589.57)	231.84 ± 275.03 (6.78–1074.10)	0.006*
SUVmax	14.80 ± 5.75 (4.32–25.55)	12.07 ± 5.89 (5.28–29.55)	0.061
SUVmean	5.54 ± 2.30 (2.21–10.48)	5.24 ± 2.13 (2.04–9.94)	0.651

* *p* < 0.05: significant difference.

### 3.4 Relationship Between Total Alteration Number in ctDNA and Metabolic Tumor Burden

In the ctDNA‐positive group, 8 patients (17%) had 1 alteration, 10 patients (21.3%) had 2 alterations, 7 patients (14.9%) had 3 alterations, and 1 patient each (2.1%) had 4 or 5 alterations. The average total alteration number in ctDNA was 1.23 ± 1.32 (range: 0–5). There was a moderate correlation between the total alteration number in ctDNA and WB‐MTV (*r* = 0.563, *p* < 0.001) and WB‐TLG (*r* = 0.459, *p* = 0.001). However, there was no significant correlation between the total alteration number in ctDNA and SUVmax (*r* = 0.249, *p* = 0.091) or SUVmean (*r* = 0.038, *p* = 0.799). When the group without detectable ctDNA was excluded, the average total alteration number in ctDNA was 2.15 ± 1.03 (range: 1–5). In this subgroup, there was no significant correlation between the total alteration number in ctDNA and SUVmax, SUVmean, or WB‐TLG (*r* = 0.007, *p* = 0.972; *r* = −0.072, *p* = 0.722; *r* = 0.305, *p* = 0.122, respectively). However, the significant correlation between the total alteration number in ctDNA and WB‐MTV (*r* = 0.500, *p* = 0.008) persisted.

### 3.5 Correlation Between VAF Values and Metabolic Tumor Burden

**FIGURE 4:**
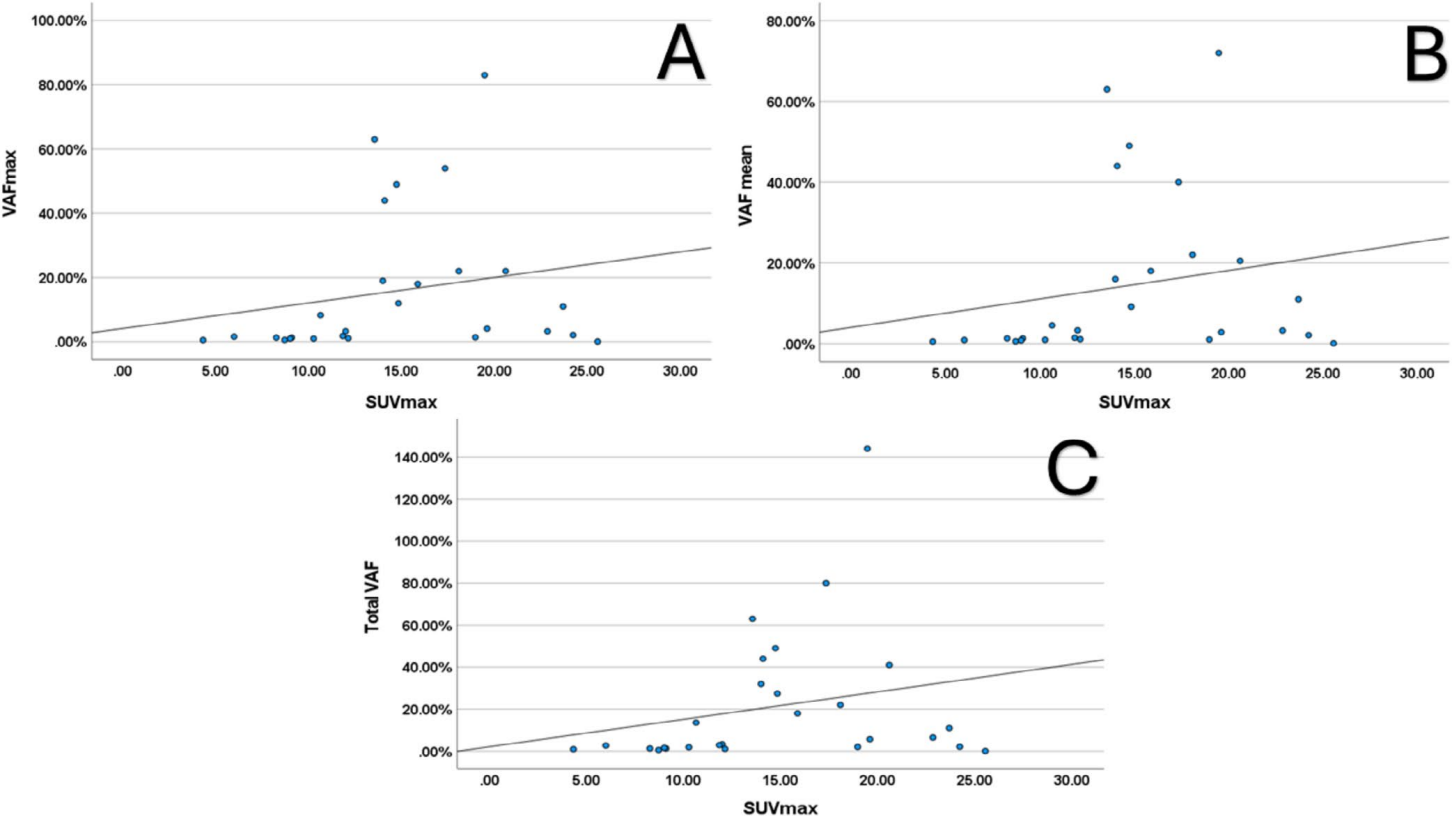
When the group without detectable ctDNA was excluded, scatter plots demonstrated the correlation between maximum variant allele frequency (VAFmax) (A), mean VAF (B), total VAF (C), and SUVmax values (*r* = 0.413, *p* = 0.032; *r* = 0.403, *p* = 0.037; and *r* = 0.377, *p* = 0.053, respectively). Although there was a weak correlation between total VAF and SUVmax, it was not statistically significant.

There was a moderate correlation between maximum VAF and SUVmax, WB‐MTV, and WB‐TLG (*r* = 0.407, *p* = 0.005; *r* = 0.457, *p* = 0.001; *r* = 0.415, *p* = 0.004, respectively). Similarly, mean VAF was moderately correlated with SUVmax, WB‐MTV, and WB‐TLG (*r* = 0.406, *p* = 0.005; *r* = 0.446, *p* = 0.002; *r* = 0.404, *p* = 0.005, respectively). The total VAF also showed moderate correlations with SUVmax, WB‐MTV, and WB‐TLG (*r* = 0.394, *p* = 0.006; *r* = 0.465, *p* = 0.001; *r* = 0.430, *p* = 0.003, respectively). There was no significant correlation between SUVmean and maximum VAF (*r* = 0.144, *p* = 0.336), mean VAF (*r* = 0.141, *p* = 0.346), or total VAF (r = 0.134, *p* = 0.370). The correlations between VAF values and metabolic parameters were similar across different VAF measures. When the group without detectable ctDNA was excluded, moderate correlations between maximum VAF and mean VAF with SUVmax remained significant (*r* = 0.413, *p* = 0.032; *r* = 0.403, *p* = 0.037). There was also a low‐level correlation between total VAF and SUVmax, but it was not statistically significant (*r* = 0.377, *p* = 0.053) (Figure 4). There was no significant correlation between VAF values and WB‐MTV, WB‐TLG, or SUVmean (Table 4).

**TABLE 4:** Spearman correlation analysis between SUV parameters (SUVmax, SUVmean, WB‐MTV and WB‐TLG), metabolic tumor burden, and circulating tumor DNA (ctDNA) metrics (VAFmax, mean VAF, total VAF, total alteration number in ctDNA).

	Patients without detected ctDNA				Patients with detected ctDNA			
	SUVmax	SUVmean	WB‐MTV	WB‐TLG	SUVmax	SUVmean	WB‐MTV	WB‐TLG
VAFmax								
Correlation coefficient	0.407**	0.144	0.452**	0.415**	0.413*	0.279	0.197	0.173
Sig. (2‐tailed)	0.005*	0.336	0.001*	0.004*	0.032*	0.159	0.324	0.389
*N*	47	47	47	47	27	27	27	27
VAFmean								
Correlation coefficient	0.406**	0.141	0.446**	0.404**	0.403*	0.269	0.176	0.143
Sig. (2‐tailed)	0.005*	0.346	0.002*	0.005*	0.037*	0.175	0.379	0.477
*N*	47	47	47	47	27	27	27	27
Total VAF								
Correlation coefficient	0.394**	0.134	0.465**	0.430**	0.377	0.247	0.245	0.221
Sig. (2‐tailed)	0.006*	0.370	0.001*	0.003*	0.053	0.215	0.219	0.268
*N*	47	47	47	47	27	27	27	27
Total alteration number in ctDNA								
Correlation coefficient	0.249	0.038	0.563**	0.459**	0.007	−0.072	0.500**	0.305
Sig. (2‐tailed)	0.091	0.799	0.000*	0.001*	0.972	0.722	0.008*	0.122
*N*	47	47	47	47	27	27	27	27

* *p* < 0.05: significant different.

** Correlation coefficient 𝜌 shows moderate correlation.

## 4 Discussion

Predicting treatment outcomes in ABC is of great importance. Information such as tumor aggressiveness, potential clinical benefits from treatment, toxicity risks, and survival depending on whether treatment is given can guide the decision‐making process between the patient and the physician. The combined use of molecular imaging methods and liquid biopsy can assist the clinician in making this assessment. Studies that use these two different modalities together for prognosis prediction, treatment response determination, and recurrence investigation have been published for many types of cancers, including breast cancer [20, 21, 22, 23]. However, the majority of previously published studies are prospective and include treatment‐naïve patients. In contrast, our study explores whether the correlation between metabolic tumor burden and ctDNA analysis in ABC can be assessed independently of treatment status.

Previous studies in literature have shown the correlation between liquid biopsy and molecular imaging methods in breast cancer [24, 25]. However, in these studies, CTCs are used as the analyte in liquid biopsy analyses. Despite the many advantages CTCs offer as an analyte, ctDNA has come to the forefront in disease monitoring due to the difficulties in isolating CTCs [8]. A recent study examined the correlation between radiologically detected tumor volume and VAF values in ABC and found a positive correlation between high tumor volume and VAF, even though VAF was not directly correlated with tumor volume [26]. Notably, our study is the first to investigate the correlation between metabolic parameters and ctDNA‐related parameters in ABC.

In this study, it was observed that WB‐MTV and WB‐TLG values were higher in patients with ctDNA detected than in those without ctDNA. There was no statistically significant relationship between SUVmax and SUVmean values and ctDNA detection. Similar studies demonstrating this relationship in other cancers have been published before [16, 17, 18]. Our study, however, is the first to demonstrate this relationship in ABC. Although these volumetric metabolic parameters are not widely used in clinical practice due to measurement difficulties and lack of standardization, a meta‐analysis published in 2020 showed that volumetric parameters like MTV and TLG are quite successful in predicting prognosis in ABC [15]. Our study suggests that integrating metabolic volumetric parameters with liquid biopsy in diagnostic, prognostic, and treatment response algorithms could enhance their effectiveness [27]. This correlation may help develop a more cost‐effective and efficient diagnostic strategy, where patients with elevated metabolic volumetric parameters on FDG PET/CT are prioritized for liquid biopsy testing. Such an approach could be particularly beneficial in resource‐limited settings or healthcare systems with restricted access to next‐generation sequencing technologies. This correlation could be leveraged to develop a more cost‐effective and efficient diagnostic algorithm, where patients with elevated metabolic volumetric parameters on FDG PET/CT could be prioritized for liquid biopsy testing. Such an approach would be particularly valuable in resource‐limited settings or healthcare systems with restricted access to next‐generation sequencing technologies.

In previous studies, patients with multiple SNVs had their VAF values analyzed by using total VAF, mean VAF, and maximum VAF to reflect the disease [18, 28]. In this study, we aimed to evaluate the correlation between these VAF values and metabolic parameters by using all these VAF values, and we performed the evaluation in two different ways. In the first evaluation, we included the group with no ctDNA detected, meaning patients with no SNV values and thus VAF values of 0. In this case, a moderate and significant correlation was observed between total VAF, mean VAF, and maximum VAF values and SUVmax, WB‐MTV, and WB‐TLG, with no significant correlation observed with SUVmean. A noteworthy point is that the correlation between all three VAF values and the metabolic parameters was similar. Studies have shown correlations between metabolic tumor burden and ctDNA levels in non‐small cell lung cancer and melanoma [29, 30], as well as between maximum VAF and ctDNA in head and neck cancers [18]. However, in these studies, no correlation was found between SUVmax and VAF values. Nonetheless, there are also studies in the literature that show a correlation between SUVmax and ctDNA levels or maximum VAF [31, 32, 33].

In the second evaluation, the group with no ctDNA detected was excluded. In this assessment, the previously observed correlation between VAF values and WB‐MTV and WB‐TLG disappeared. However, the moderate correlation between maximum VAF and mean VAF values and SUVmax persisted. The correlation between total VAF and SUVmax weakened and fell below statistically significant levels. These results suggest that the relationship between VAF and WB‐MTV and WB‐TLG is primarily driven by the presence of ctDNA. When this relationship is disrupted, the correlation with tumor burden disappears. However, the persistence of the correlation between SUVmax and VAF indicates that this relationship is directly associated with high VAF values. The established relationship between VAF values and SUVmax, a parameter known to be associated with aggressive disease, suggests that an increase in VAF may also be linked to aggressive disease [34]. While the association between VAF status and poor prognosis has been previously reported, our study suggests that this relationship may also be independently established in ABC patients who continue to progress regardless of their treatment status [26]. Additionally, it should be noted that maximum VAF and mean VAF show a stronger correlation with SUVmax. Similar findings have been observed in two studies on non‐small cell lung cancer, where no significant relationship was found between cfDNA levels and metabolic volumetric parameters [32, 35]. However, it should be considered that these studies used cfDNA, and the sample sizes were relatively small. Although metabolic volumetric parameters were not used, Zhong et al. in 2024 concluded in their study on ABC that there was no direct correlation between tumor volume and VAF [26].

A study published in 2020 showed the relationship between the total alteration number in ctDNA and prognostic factors [11]. However, to our knowledge, there is no study that examines the relationship between total alteration number in ctDNA and metabolic tumor burden. Our study is the first publication to investigate this relationship. When the relationship between total alteration number in ctDNA and metabolic parameters was examined, a strong correlation was observed with WB‐MTV both when the group without ctDNA detection was included and excluded. However, the correlation observed with WB‐TLG in the first group disappeared in the second group. This suggests that ctDNA Total Change may be related to tumor volume independently of metabolic parameters. Nevertheless, the need for further studies on this topic is evident. The results of our study suggest that ctDNA Total Change could be a valuable tool in prognosis and treatment response assessment.

There are certain limitations to this study. Some of the patients included in the study were diagnosed as far back as 2005, and therefore, some archival documents related to their treatments were incomplete. As a result, information about previous treatments for the patients is not included in this article. Additionally, there was a relatively long time between FDG PET/CT and liquid biopsy tests in some patients. However, only 5 patients had a gap longer than 30 days, and most patients (29/47) had their tests performed within less than 15 days of each other. Due to the advanced stage of the disease in the patients included in the study, molecular profiling could not be performed via solid tissue biopsy for comparative evaluation with liquid biopsy findings.

Considering the data obtained from our study, we suggest that FDG PET/CT imaging could assist in selecting patients with a high probability of positive liquid biopsy as the first step in incorporating molecularly targeted therapies. Especially, metabolic parameters may provide guidance, independent of the patient's treatment status. The combined use of ctDNA detection and FDG PET/CT could contribute to prognostic and predictive evaluations during both the initial diagnosis and follow‐up stages after definitive treatment in breast cancer. However, further studies are needed on this topic. Another point highlighted by our study is that the total alteration number in ctDNA may be used as an independent parameter in diagnosis, post‐treatment assessment, and prognosis. It could be suggested that this parameter, being closely related to WB‐MTV, could serve as a tool for reflecting the volumetric extent of the disease. Of course, further studies are needed to make a definitive conclusion. These studies should also focus on different types of cancer and pre‐treatment assessments.

## 5 Conclusion

This study found that metabolic tumor burden was higher in patients with ctDNA detection. We observed that the correlation between VAF values and metabolic volumetric parameters was largely related to the presence of ctDNA, and this correlation disappeared when the group without ctDNA detection was excluded. However, the positive correlation with SUVmax in the same group remained significant, albeit weakened. It was also observed that the ctDNA Total Change was correlated with metabolic parameters, and this correlation was maintained with WB‐MTV even when the group without ctDNA detection was excluded.

## Author Contributions

**Recep Halit Tokac:** writing – original draft, methodology, investigation, resources, data curation, conceptualization, formal analysis. **Ekin Cemre Bayram Tokac:** methodology, visualization, writing – original draft, data curation, resources, investigation. **Ulkem Yararbas:** writing – review and editing, supervision. **Ayca Aykut:** methodology, supervision. **Asude Durmaz:** methodology, supervision. **Haluk Akin:** supervision. **Aziz Murat Argon:** writing – review and editing, supervision, project administration.

## Ethics Statement

This study was approved by the Institutional Ethics Committee of the Ege University Faculty of Medicine Hospital (Approval No: 24‐7 T/34). All methods were conducted in accordance with the ethical standards of the 1964 Declaration of Helsinki and its later amendments or comparable ethical standards.

## Conflicts of Interest

The authors declare no conflicts of interest.

## Supporting information


**Table S1.** The detected ctDNA alterations, VAF values, and reading depth/quality scores in patients.
**Table S2.** The liquid biopsy and FDG PET/CT parameters of HR+, HER2−, HER2+, and triple‐negative patients.

## Data Availability

The datasets generated and/or analyzed during the current study are available from the corresponding author on reasonable request.
