# Supplementary material for: Cross‐Sectional Analysis of Metabolic Tumor Burden Detected by F‐18 FDG PET/CT and Circulating Tumor DNA in Advanced Breast Cancer
Source: Cancer Med. 2025 Aug 18;14(16):e71049. doi: 10.1002/cam4.71049 (PMC12359722; doi:10.1002/cam4.71049)
Supplement: Supplementary file 1 — Table S1. The detected ctDNA alterations, VAF values, and reading depth/quality scores in patients. Table S2. The liquid biopsy and FDG PET/CT parameters of HR+, HER2−, HER2+, and triple‐negative patients. [file CAM4-14-e71049-s001.docx]

**Supplementary Information**

**Suplementary Table 1:** Table shows the detected ctDNA alterations, VAF values, and reading depth/quality scores in patients.

| Patient  Number | Gene | Variant | VAF | Read Depth* |
| --- | --- | --- | --- | --- |
| 1 | TP53 | c.993+2T>C | 0.57% | 4383 |
|  | TP53 | c.919+1G>T | 0.51% | 3960 |
|  | PTEN | p.N323fs | 1.6% | 1969 |
| 2 | PIK3CA | p.H1047R | 0.57% | 5074 |
|  | ESR1 | p.Y537S | 0.63% | 6012 |
| 3 | RB1 | p.R251* | 4.3% | 4638 |
|  | RB1 | p.Q504* | 1.0% | 4313 |
|  | TP53 | p.V274F | 8.3% | 4320 |
|  | MET amp. |  |  | 2,16 |
| 4 | TP53 | p.V274F | 1.3% | 2982 |
|  | MET amp. |  |  | 2,21 |
|  | EGFR amp. |  |  | 3,00 |
| 5 | TP53 | p.M133K | 4.8% | 2773 |
|  | AKT1 | p.E17K | 13% | 5571 |
| 6 | PIK3CA | p.H1047R | 0.11% | 4313 |
|  | EGFR amp. |  |  | 2,43 |
|  | MET amp. |  |  | 3,26 |
| 7 | TP53 | p.R342* | 0.54% | 4735 |
|  | PIK3CA | p.E545K | 26% | 7750 |
| 8 | ESR1 | p.L536R | 3.4% | 3427 |
|  | PIK3CA | p.Q546E | 12% | 4317 |
|  | PIK3CA | p.H1047R | 12% | 3298 |
|  | EGFR amp. |  |  | 4,59 |
|  | MET amp. |  |  | 4,42 |
| 9 | BRAF | p.V600E | 1.1% | 7426 |
|  | PMS2 | c.2174+1G>A | 1.8% | 3009 |
| 10 | TP53 | p.R248G | 0.09% | 6836 |
| 11 | CDKN2A | p.R80* | 0.63% | 1338 |
|  | EGFR amp. |  |  | 42,80 |
|  | MET amp. |  |  | 38,38 |
| 12 | ESR1 | p.D538G | 0.19% | 5437 |
|  | PIK3CA | p.E545K | 22% | 7229 |
|  | EGFR amp. |  |  | 14.98 |
| 13 | RB1 | p.S834* | 0.19% | 3522 |
|  | ESR1 | p.D538G | 13% | 2633 |
| 14 | PIK3CA | p.N345K | 2.1% | 2473 |
| 15 | TP53 | p.H179P | 0.18% | 2596 |
| 16 | VHL | p.R161* | 1.3% | 3253 |
| 17 | ESR1 | p.L536R | 0.99% | 7907 |
|  | ESR1 | p.D538G | 0.62% | 7305 |
| 18 | ESR1 | p.E380Q | 22% | 11895 |
|  | ERBB2 amp. |  |  | 10,56 |
| 19 | JAK2 | p.V617F | 0.1% | 2504 |
| 20 | ESR1 | p.D538G | 3.3% | 2891 |
| 21 | CCND3 | p.R167W | 0.49% | 7305 |
| 22 | PIK3CA | p.Q546K | 0.88% | 5246 |
|  | KRAS | p.G12V | 1.0% | 4860 |
| 23 | RB1 | c.1215+1G>A | 61% | 3175 |
|  | PIK3CA | p.Q546K | 83% | 2861 |
| 24 | TP53 | p.Y220H | 0.44% | 6964 |
|  | ERBB2 amp. |  |  | 17,34 |
|  | EGFR amp. |  |  | 42,64 |
| 25 | TP53 | p.Q331* | 0.63% | 4419 |
|  | PIK3CA | p.E545K | 1.4% | 3857 |
| 26 | TP53 | p.E545K | 3.2% | 7409 |
|  | TP53 | p.R282W | 3.3% | 8078 |
|  | EGFR amp. |  |  | 3,10 |
| 27 | ESR1 | p.D538G | 0.54% | 3025 |
|  | PIK3CA | p.E545K | 0.42% | 4315 |

*In amplifications, the quality score criterion was mentioned instead of reading depth.

|  | **Number of Patients** | **ctDNA + Patients** | **Total Number of Alterations** | **ctDNA Alterations** | **SUVmax** | **SUVmean** | **WB-MTV** | **WB-TLG** | **VAFmax** | **VAFmean** | **Total VAF** |
| --- | --- | --- | --- | --- | --- | --- | --- | --- | --- | --- | --- |
| **HR +, HER2 -** | 32 | 18 (56.3%) | 1,22 ± 1,34  (0-5) | PIK3CA(n=9), TP53(n=4), ESR1(n=4), CDKN2A(n=1), PTEN(n=1), EGFR(n=1), RB1(n=1), PMS2(n=1), VHL(n=1), JAK2(n=1), CCND3(n=1), KRAS(n=1), BRAF(n=1), EGFR amp.(n=4), MET amp.(n=4) | 13,45 ± 6,18  (4,32-29,55) | 5,32 ± 2,35  (2,04-10,48) | 175,83 ± 289,64  (0,95-1444,39) | 851,38 ± 1505,50  (6,78- 6589,57) | 8,03% ± 19,65%  (0,00%- 83,00%) | 7,54% ± 18,36%  (0,00%- 72,00%) | 10,67% ± 28,34%  (0,00%- 144,00%) |
| **HER2 +** | 12 | 7 (58.3%) | 1,33 ± 1,43  (0-4) | TP53(n=4), ESR1(n=3), RB1(n=2), PIK3CA(n=1), AKT1(n=1), EGFR amp.(n=2), MET amp.(n=1), ERBB2 amp.(n=1) | 13,37 ± 4,51  (5,35-20,40) | 5,25 ± 1,68  (3,09-8,91) | 109,09 ± 187,78  (1,19-648,62) | 569,99 ± 979,14  (7,73-3308,92) | 9,89% ± 13,58%  (0,00%- 44,00%) | 9,09% ± 13,42%  (0,00%- 44,00%) | 13,13% ± 16,85%  (0,00%- 44,00%) |
| **Triple Negative** | 3 | 2 (66.6%) | 1,00 ± 1,00  (0-2) | TP53(n=2), PIK3CA(n=1) | 16,71 ± 9,17  (7,25-25,55) | 6,99 ± 2,65  (4,13-9,36) | 42,60 ± 51,91  (11,15-102,52) | 370,72 ± 511,58  (46,01- 960,43) | 18,03% ± 31,15%  (0,00%- 54,00%) | 13,63% ± 23,07%  (0,00%- 40,00%) | 26,70% ± 46,16%  (0,00%- 80,00%) |

**Suplementary Table 2:** Table shows the liquid biopsy and FDG PET/CT parameters of HR+, HER2-, HER2+, and Triple Negative patients.
